# Specific static and dynamic functional network connectivity changes in thyroid-associated ophthalmopathy and it predictive values using machine learning

**DOI:** 10.3389/fnins.2024.1429084

**Published:** 2024-08-23

**Authors:** Hao Liu, Yu-Lin Zhong, Xin Huang

**Affiliations:** ^1^School of Ophthalmology and Optometry, Jiangxi Medical College, Nanchang University, Nanchang, Jiangxi, China; ^2^Department of Ophthalmology, Jiangxi Provincial People’s Hospital, The First Affiliated Hospital of Nanchang Medical College, Nanchang, Jiangxi, China

**Keywords:** thyroid-associated ophthalmopathy, independent component analysis, resting-state network, functional network connectivity, functional connectivity

## Abstract

**Background:**

Thyroid-associated ophthalmopathy (TAO) is a prevalent autoimmune disease characterized by ocular symptoms like eyelid retraction and exophthalmos. Prior neuroimaging studies have revealed structural and functional brain abnormalities in TAO patients, along with central nervous system symptoms such as cognitive deficits. Nonetheless, the changes in the static and dynamic functional network connectivity of the brain in TAO patients are currently unknown. This study delved into the modifications in static functional network connectivity (sFNC) and dynamic functional network connectivity (dFNC) among thyroid-associated ophthalmopathy patients using independent component analysis (ICA).

**Methods:**

Thirty-two patients diagnosed with thyroid-associated ophthalmopathy and 30 healthy controls (HCs) underwent resting-state functional magnetic resonance imaging (rs-fMRI) scanning. ICA method was utilized to extract the sFNC and dFNC changes of both groups.

**Results:**

In comparison to the HC group, the TAO group exhibited significantly increased intra-network functional connectivity (FC) in the right inferior temporal gyrus of the executive control network (ECN) and the visual network (VN), along with significantly decreased intra-network FC in the dorsal attentional network (DAN), the default mode network (DMN), and the left middle cingulum of the ECN. On the other hand, FNC analysis revealed substantially reduced connectivity intra- VN and inter- cerebellum network (CN) and high-level cognitive networks (DAN, DMN, and ECN) in the TAO group compared to the HC group. Regarding dFNC, TAO patients displayed abnormal connectivity across all five states, characterized by notably reduced intra-VN connectivity and CN connectivity with high-level cognitive networks (DAN, DMN, and ECN), alongside compensatory increased connectivity between DMN and low-level perceptual networks (VN and basal ganglia network). No significant differences were observed between the two groups for the three dynamic temporal metrics. Furthermore, excluding the classification outcomes of FC within VN (with an accuracy of 51.61% and area under the curve of 0.35208), the FC-based support vector machine (SVM) model demonstrated improved performance in distinguishing between TAO and HC, achieving accuracies ranging from 69.35 to 77.42% and areas under the curve from 0.68229 to 0.81667. The FNC-based SVM classification yielded an accuracy of 61.29% and an area under the curve of 0.57292.

**Conclusion:**

In summary, our study revealed that significant alterations in the visual network and high-level cognitive networks. These discoveries contribute to our understanding of the neural mechanisms in individuals with TAO, offering a valuable target for exploring future central nervous system changes in thyroid-associated eye diseases.

## 1 Introduction

Thyroid-associated ophthalmopathy (TAO), also recognized as Graves’ ophthalmopathy, stands as the prevailing autoimmune orbital condition in adults. Individuals afflicted with TAO typically exhibit periorbital edema, upper eyelid retraction, diplopia, exophthalmos, and compromised visual acuity as primary clinical features (Bahn, 2010; Maheshwari and Weis, 2012; McAlinden, 2014). The etiology of TAO remains uncertain, yet it is commonly associated with the breakdown of immune tolerance toward the thyrotropin receptor (TSHR) and irregular levels of thyrotropin receptor antibodies (TRAbs) (McLachlan and Rapoport, 2014; Diana et al., 2021; Nicolì et al., 2021). Several studies have indicated the potential involvement of the insulin-like growth factor 1 (IGF-1) receptor in the pathogenesis of TAO (Smith et al., 2017; Smith and Janssen, 2019). Risk factors for TAO include smoking, thyroid dysfunction, radioactive iodine therapy, and selenium deficiency (Bartalena et al., 2020; Cao et al., 2022; Yu et al., 2022). The natural progression of TAO can be categorized into an active phase marked by acute inflammatory reactions and distinctive ocular symptoms, and an inactive phase characterized by fibrosis (Weiler, 2017). With an enhanced comprehension of TAO, it has become evident that beyond ocular manifestations, TAO can potentially induce alterations in the brain and is closely linked to central nervous system symptoms, including cognitive impairment (Stern et al., 1996; Bunevicius and Prange, 2006; Vogel et al., 2007; Silkiss and Wade, 2016; Bruscolini et al., 2018). Hence, investigating the neural mechanisms underlying TAO is crucial for advancing disease management and enhancing the quality of life for affected individuals.

Previous neuroimaging investigations have consistently revealed structural and functional brain changes in individuals with TAO, believed to correlate with visual deficits and central nervous system manifestations. Structural brain changes related to vision and cognition are present in TAO patients. Some studies have found altered gray matter thickness or volume in the occipital and frontal lobes in patients with TAO (Silkiss and Wade, 2016; Luo et al., 2022), and significantly increased iron deposition in brain regions corresponding to visual and cognitive deficits (Hu et al., 2023). Diffusion tensor imaging has shown reduced fractional anisotropy (FA) in the right superior occipital gyrus and middle occipital gyrus in patients with TAO, and FA values are positively correlated with visual acuity (Wu et al., 2020). The topological organization of the structural network of the brain is also disrupted in TAO patients, which may be related to clinical psychiatric dysfunction (Wu et al., 2021). In addition, patients with TAO exhibit functional brain abnormalities. Wen et al. (2023) found that abnormal dynamic amplitude of low-frequency fluctuation (dALFF) was present in the visual cortex of TAO patients. Jiang et al. (2022) reported altered functional connectivity density (FCD) values in the prefrontal cortex of TAO patients. Local brain functional connectivity as well as local temporal variability of brain activity in brain regions associated with vision and cognition are also altered in TAO patients (Wen et al., 2022; Jiang et al., 2023). This series of studies found that TAO mainly leads to changes in brain regions such as the superior occipital gyrus, middle occipital gyrus, fusiform gyrus, and cuneus lobe, which are related to visual function. At the same time, abnormalities were also found in frontal and parietal regions such as the middle frontal gyrus, precuneus, cingulate gyrus, post-central gyrus, and superior parietal lobe, which are involved in cognition-related functions.

Previous research has concentrated on localized brain function and structural irregularities in TAO patients. Our brain function is governed by the coordinated activities of various brain networks. However, how static and dynamic large-scale brain network functional connectivity is altered in TAO patients remains unclear. Thus, investigating the TAO brain network in our study can enhance comprehension of the neural mechanisms underlying TAO damage. The human brain is a sophisticated and dynamic system that exhibits nonstationary neural activity and swiftly changing neural interactions. Inherently dynamic, human brain activity is characterized by temporal variability, which influences the functional capabilities of neural networks. Fluctuations in blood oxygen level-dependent (BOLD) signals during the resting state reflect the baseline neuronal activity in the brain. These low-frequency fluctuations correspond to functionally relevant resting-state networks (RSNs) (Damoiseaux et al., 2006; Smith et al., 2009). ICA has been utilized as a powerful data-driven method to identify RSNs, encompassing low-level perceptual networks (visual, auditory, and sensorimotor) as well as high-level cognitive networks (default mode, executive control, and salience). This approach unveils various patterns of interactions within the human brain (van de Ven et al., 2004; Beckmann et al., 2005; De Luca et al., 2006). ICA technology can extract distinct functional brain networks from intricate brain imaging data without requiring predetermined assumptions. It offers high resolution and is adaptable for various brain research and data analysis tasks, showcasing exceptional flexibility and versatility. It has been previously applied to the study of several ophthalmic diseases such as strabismus (Jin et al., 2022), diabetic retinopathy (Huang et al., 2020), congenital blindness (Wang et al., 2014), and neuromyelitis optica (Yang et al., 2022), but it has not yet been used in the study of TAO. FNC can capture the temporal correlations between RSNs, but static FNC relies on the assumption that functional interactions remain consistent throughout resting-state scanning. An increasing body of research indicates that brain functional connectivity is dynamic, exhibiting significant time-varying characteristics (Hutchison et al., 2013a; Damaraju et al., 2014; Liégeois et al., 2017). Dynamic FNC can reflect transient and periodic whole-brain temporal coupling patterns (Wens et al., 2019), and offer a more comprehensive and profound understanding of the neural mechanisms of disease by examining data that is not accessible through sFNC. In addition, classification based on neuroimaging features using machine learning has been widely used in various fields and has shown good classification performance (Davatzikos et al., 2005; Wang et al., 2007; Li et al., 2022; Xu et al., 2022).

Therefore, this study utilized ICA to investigate both static and dynamic alterations in functional network connectivity among individuals with TAO. Initially, RSNs were identified through ICA, followed by a comparison of intra-network functional connectivity and differences in FNC between the TAO group and the control group. Subsequently, a combination of sliding window and k-mean cluster analysis was employed to examine dFNC changes in TAO patients and to analyze differences in three dynamic temporal metrics between the two cohorts. Lastly, an attempt was made to classify TAO patients and HCs by utilizing SVM based on FC and FNC data. It was hypothesized that TAO patients would exhibit anomalous static and dynamic functional network connectivity.

## 2 Materials and methods

### 2.1 Participants

Thirty-two individuals with TAOand 30 HCs, matched for sex, age, and education, were recruited from the Department of Ophthalmology at Jiangxi Provincial People’s Hospital for this study. The diagnosis of TAO in all patients was made by two experienced ophthalmologists in accordance with the diagnostic criteria set by the American Academy of Ophthalmology (Bartley and Gorman, 1995). Furthermore, their visual acuity, visual fields, color vision, and pupillary reflexes were assessed. The disease activity of TAO was determined using the modified 7-point Mourits’ Clinical Activity Score (CAS) (Bartalena et al., 2016). Patients with CAS ≥ 3 were included in the active TAO group; otherwise, they were included in the inactive group.

The inclusion criteria for subjects were: (1) absence of contraindications to Magnetic resonance imaging (MRI) scanning (such as no pacemakers or implanted metal devices); (2) no claustrophobia; and (3) no history of heart or brain disease.

The exclusion criteria for all subjects were as follows: (1) presence of other ocular diseases (such as vitreous hemorrhage, high myopia, optic neuritis, retinal degeneration, amblyopia, strabismus, glaucoma, cataracts, etc.); (2) a history of traumatic eye injuries or ophthalmological surgeries; (3) a history of neurological or psychiatric disorders (including craniocerebral trauma, bi-directional affective disorders, schizophrenia, etc.); and (4) alcohol or drug abuse.

The study protocol was in compliance with the Declaration of Helsinki and obtained approval from the Research Ethics Committee of Jiangxi Provincial People’s Hospital. Prior to their participation in the study, all participants were briefed on the study’s objectives, procedures, and potential risks, and they provided written informed consent to take part.

### 2.2 MRI acquisition

MRI scans were conducted using a 3-Tesla scanner (Discovery MR 750W system; GE Healthcare, Milwaukee, WI, United States) equipped with an eight-channel head coil. Functional images were acquired using a gradient-echo-planar imaging sequence. Subjects were directed to remain in a resting state with their eyes closed, stay relaxed without focusing on any specific thoughts, and avoid falling asleep. Whole-brain T1-weighted images were obtained using a three-dimensional brain volume imaging (3D-BRAVO) MRI protocol. T1 following parameters: repetition time (TR)/echo time (TE) = 8.5/3.3, thickness = 1.0 mm, no intersection gap, acquisition matrix = 256 × 256, field of view = 240 mm^2^ × 240 mm^2^, and flip angle = 12°. Functional images were obtained by using a gradient echoplanar imaging sequence with the following parameters: TR/TE = 2,000 ms/25 ms, thickness = 3.0 mm, gap = 1.2 mm, acquisition matrix = 64 × 64, flip angle = 90°, field of view = 240 mm^2^ × 240 mm^2^, voxel size = 3.6 mm^3^ × 3.6 mm^3^ × 3.6 mm^3^, and 35 axial slices. All the subjects were instructed to rest quietly with their eyes closed and relaxed without thinking about anything in particular or falling asleep.

### 2.3 Data preprocessing

All pre-processing was performed using the toolbox for Data Processing and Analysis of Brain Imaging (DPABI)^1^ (Yan et al., 2016), which is based on Statistical Parametric Mapping (SPM12)^2^ implemented in MATLAB 2013a (MathWorks, Natick, MA, United States) and briefly the following steps: (1) Remove the first 10 volumes. (2) Slice timing effects, motion corrected. For head motion parameters, more than 2 mm or for whom rotation exceeded 1.5° during scanning were excluded (Van Dijk et al., 2012). (3) Normalized data [in Montreal Neurological Institute (MNI) 152 space] were re-sliced at a resolution of 3 mm × 3 mm × 3 mm. (4) Spatial smoothing by convolution with an isotropic Gaussian kernel of 6 mm × 6 mm × 6 mm full width at half maximum.

### 2.4 Group ICA analysis

Group ICA was conducted to decompose the data into independent components (ICs) utilizing the Group ICA Of fMRI Toolbox(GIFT)toolbox (version 3.0b).^3^ Initially, 29 IC maps were estimated in this study by applying the minimum description length criterion to account for spatial correlation. Subsequently, the ICs for each subject were obtained through the group ICA back-reconstruction step and then transformed into z-scores (Zuo et al., 2010). Components retained for further analysis among the 29 estimated ICs were selected based on the largest spatial correlation with specific RSN templates (Shirer et al., 2012; Wang et al., 2014). The IC time courses and spatial maps for each subject were transformed to z-scores. Seventeen RSNs were identified in this study. We identified 17 significant independent components (ICs) based on the following criteria: (a) peak coordinates of spatial maps predominantly located in gray matter, (b) absence of spatial overlap with vascular, ventricular, or susceptibility artifacts, and © time courses characterized by predominantly low-frequency signals (with a ratio of powers below 0.1 Hz to 0.15–0.25 Hz in the frequency spectrum). The identified resting-state networks (RSNs) included the dorsal attention network (DAN) represented by IC6 and IC15, the auditory network (AN) by IC7, the default mode network (DMN) by IC9, IC14, IC18, IC27, and IC28, the salience network (SN) by IC10, the executive control network (ECN) by IC11 and IC17, the visual network (VN) by IC12, IC13, and IC25, the sensorimotor network (SMN) by IC21, the cerebellum network (CN) by IC24, and the basal ganglia network (BGN) by IC26.

### 2.5 Static functional network connectivity analysis

#### 2.5.1 Intra-network functional connectivity analysis

The intra-network FC was expressed as the z-score of each voxel, indicating the extent to which the time series of a particular voxel correlates with the mean time series of its associated component.

#### 2.5.2 Inter-network functional connectivity analysis

FNC analysis was conducted utilizing the MANCOVAN toolbox within the GIFT software to investigate alterations in the predefined 17 spatial independent component (IC) pairs of functional connections. Initially, at the frequency range of 0.01–0.1 Hz, the selected IC underwent de-trending, de-peaking, and low-pass filtering procedures. Subsequently, the pair correlations of these ICs were computed and then transformed using Fisher’s Z-transform.

#### 2.5.3 Dynamic functional network connectivity analysis

The dFNC matrix was calculated using a sliding window method. In this study, the window width was set to a TR of 30 (60s), and the window was slide along the time axis in steps of 2 TR. The window width of 30 TRs was chosen according to previous d-FNC analyses using window sizes of 30 TRs (Abrol et al., 2017; Faghiri et al., 2018). Some studies have shown that window sizes between 30 and 60 s can capture other changes in functional connectivity not found in larger window sizes (Hutchison et al., 2013b; Allen et al., 2014; Damaraju et al., 2014).

The dynamic functional network connectivity (dFNC) matrices of all subjects underwent clustering via the k-means clustering algorithm to evaluate the frequency and configuration of recurring dFNC patterns. In this analysis, the Manhattan city distance was utilized to gauge the similarity among various time windows. To enhance the algorithm’s ability to escape local minima, the maximum number of iterations was set to 500, with 150 repetitions. The elbow rule was applied to determine the optimal number of clusters, resulting in *k* = 5. Subsequently, the dFNC matrix of all subjects was partitioned into five dFNC states, representing recurrent instantaneous FC patterns across different windows and subjects. The cluster centroid was termed as the dFNC matrix at the center of each cluster.

**FIGURE 1:**
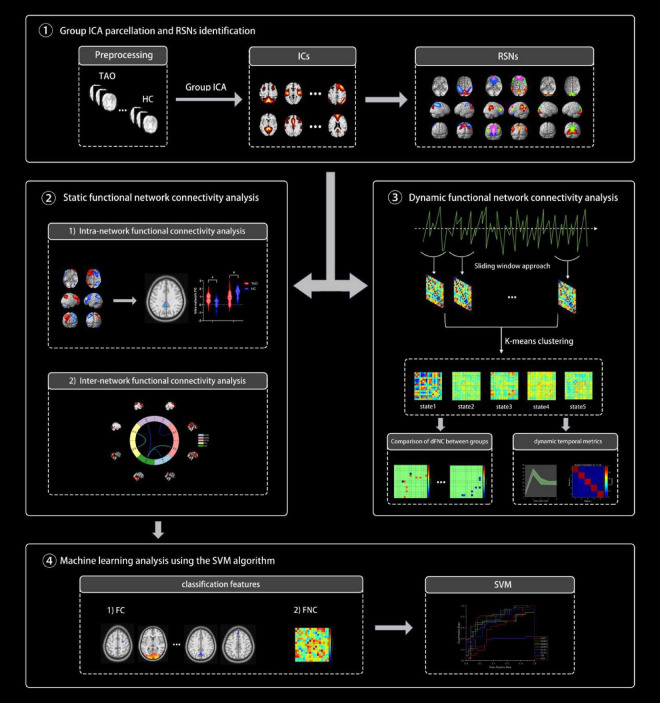
An overview of the analytical procedures: (1) After a preprocessing step, group independent component analysis (ICA) was performed and resting-state networks (RSNs) were identified. (2) Static functional connectivity differences within and between networks were calculated and compared between the two groups. (3) Combine the sliding window method and k-mean cluster analysis to identify five stable repetitive states and compare the differences in dFNC and three dynamic temporal metrics between the two groups. (4) Using FC and FNC as classification features, we attempted to use support vector machine (SVM) to classify TAO patients and healthy controls (HCs).

Various temporal characteristics were computed as follows: (i) the fraction of time, defined as the ratio of the number of time windows in a state to the total number of time windows, (ii) the mean dwell time, representing the average duration in a specific state, and (iii) the number of transitions, indicating how frequently the subject shifted from one state to another during the scan duration. An outline of the analytical processes is illustrated in Figure 1.

## 3 Statistical analysis

The ICs corresponding to seventeen RSNs were extracted from all subjects, and one-sample *t*-tests were conducted for the spatial maps of each RSN using SPM12 software. The statistical significance thresholds were established at *P* < 0.001, corrected for false discovery rate (FDR).

Two-sample *t*-tests were employed to examine the discrepancies between the two groups in the intra-network FC within RSN maps (a two-tailed approach, voxel-level significance set at *P* < 0.01, Gaussian random field correction, and cluster-level significance at *P* < 0.05). The Gaussian random field method was utilized to address multiple comparisons and covariates of age and sex were regressed using SPM12 software.

Inter-network functional connectivity analysis was conducted to assess the temporal associations between RSNs. For the significant correlation pairs, the average time lags were computed for each group, reflecting the delay between the time courses of the two correlated RSNs. Two-sample *t*-tests were performed to compare the unique temporal relationships between RSNs across the two groups (*p* < 0.05).

The disparities in dFNC between the TAO and HCs groups were evaluated using two independent samples *t*-tests within the Stats module of the GIFT software package. A significance level of *p* < 0.01 denoted a statistically significant distinction. Additionally, a two-sample *t*-test was employed to assess the contrasts in the fraction of time, mean dwell time, and number of transitions for each state between the groups, with a significance threshold of *p* < 0.05.

### 3.1 Support vector machine analysis

We performed machine learning analysis to explore whether FC and FNC are potential neuroimaging metrics for the diagnosis of TAO using SVM algorithms (Schrouff et al., 2013). The SVM classifier was then validated using leave-one-out cross-validation (LOOCV). Classification accuracy, sensitivity, and specificity were calculated, then the receiver operating characteristic (ROC) curves were generated and the corresponding area under the curve (AUC) was obtained to assess classification performance.

## 4 Results

### 4.1 Demographic and clinical characteristics

Significant differences were observed in best-corrected visual acuity (*P* < 0.001) between the two groups. However, there were no significant differences in sex, age, education, or body mass index between the two groups. Further details are provided in Table 1.

**TABLE 1:** Demographics and visual measurements between two groups.

Condition	TAO group	HC group	*t*	*p*
Gender (male/female)	18/14	14/16	N/A	N/A
Age (years)	51.03 ± 6.44	50.03 ± 5.61	0.638	0.53
Education	11.56 ± 2.75	11.47 ± 2.29	0.149	0.88
BCVA-OD	0.65 ± 0.13	1.03 ± 0.14	−10.778	< 0.001*
BCVA-OS	0.64 ± 0.15	1.05 ± 0.16	−10.055	**< 0.001** *
MoCA	25.26 ± 0.83	25.44 ± 0.77	−0.888	0.38

BCVA, best corrected visual acuity; OD, oculus dexter; OS, oculus sinister; MoCA, Montreal Cognitive Assessment; TAO, thyroid-associated ophthalmopathy; HC, health control.

*Indicate *p* < 0.001.

### 4.2 Spatial pattern of RSNs in each group

**FIGURE 2:**
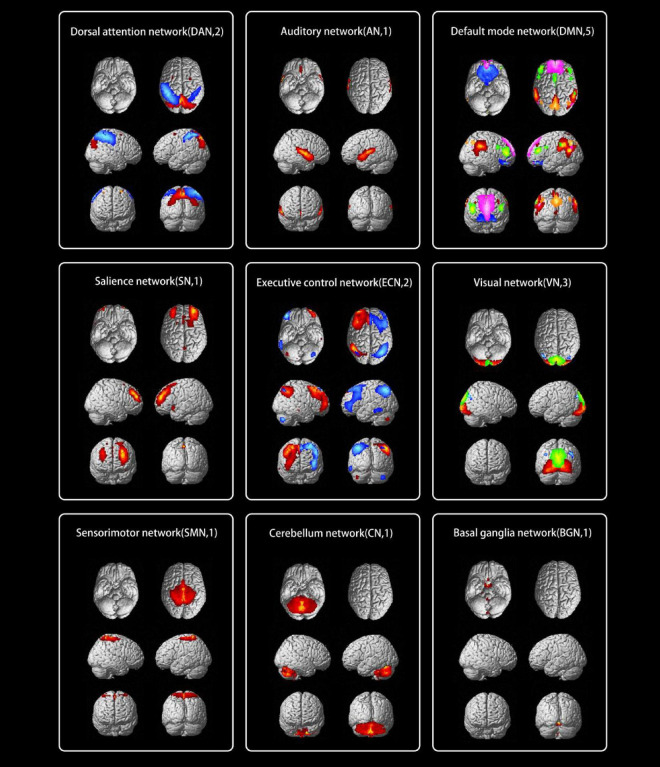
The typical spatial patterns in each RSN of both TAO and HC groups, including DAN (IC6, IC15), AN (IC7), DMN (IC9, IC14, IC18, IC27, IC28), SN (IC10), ECN (IC11, IC17), VN (IC12, IC13, IC25), SMN (IC21), CN (IC24), and BGN (IC26). Different colors pass spatial information. RSN, resting-state network; TAO, thyroid-associated ophthalmopathy; HC, health control; IC, independent component; DAN, dorsal attention network; AN, auditory network; DMN, default mode network; SN, salience network; ECN, executive control network; VN, visual network; SMN, sensorimotor network; CN, cerebellum network; BGN, basal ganglia network.

The typical spatial patterns in each RSN of both TAO and HC groups, as illustrated in Figure 2. Seventeen of these components coincided with RSNs included: dorsal attention network (DAN) (IC6, IC15), auditory network (AN) (IC7), default mode network (DMN) (IC9, IC14, IC18, IC27, IC28), salience network (SN) (IC10), executive control network (ECN) (IC11, IC17), Visual network (VN) (IC12, IC13, IC25), SMN (IC21), cerebellum network (CN) (IC24), and basal ganglia network (BGN) (IC26).

### 4.3 Altered RSNs in the TAO group

**FIGURE 3:**
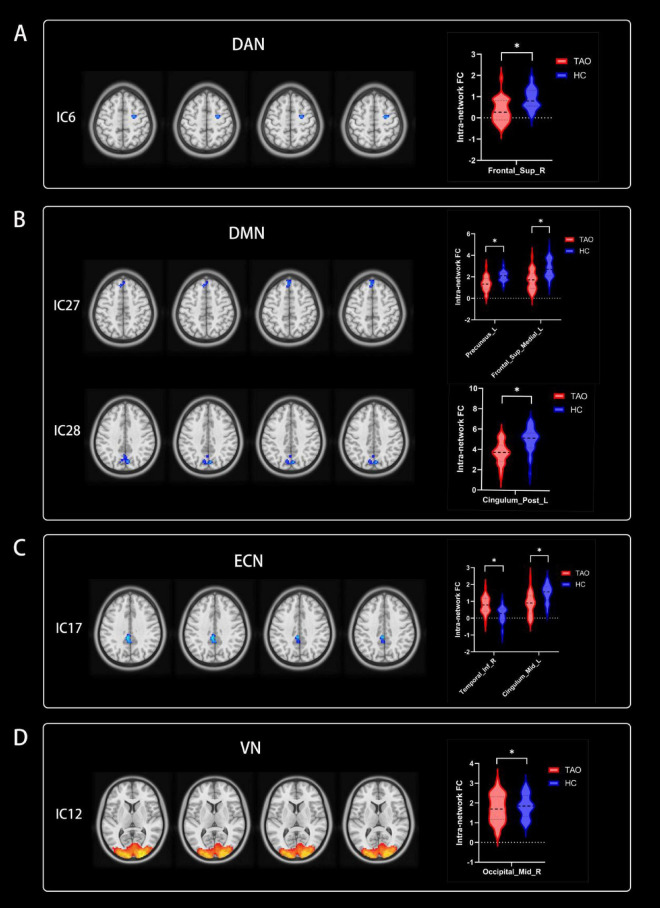
Group functional network connectivity (FNC) differences within resting-state networks (two-tailed, voxel-level *P* < 0:01; GRF correction, cluster-level *P* < 0:05). Compared with HC group, cool colors indicated the decreased functional connectivity and hot colors indicated the increased functional connectivity in the TAO group. **(A–D)** Correspond to different resting-state networks. DAN, DMN, ECN and VN. *Indicate voxel-level *P* < 0.01; GRF correction, cluster-level *P* < 0.05. RSNs, resting-state networks; TAO, thyroid-associated ophthalmopathy; HC, health control; IC, independent component; DAN, dorsal attention network; DMN, default mode network; ECN, executive control network; VN, visual network; Sup, superior; Post, posterior; Inf, inferior; Mid, middle; R, right; L, left.

Significant increases in intra-network FC within RSNs were detected in the TAO group compared to the HC group (refer to Figure 3 and Table 2). Specifically, the TAO group exhibited heightened intra-network FC in the right inferior temporal gyrus of the ECN and the right middle occipital gyrus of the VN in contrast to the HC group. Conversely, reduced intra-network FC was observed in the TAO group, manifesting in the right superior frontal gyrus of the DAN, the left precuneus, the left medial superior frontal gyrus, and the left posterior cingulum of the DMN, as well as the left middle cingulum of the ECN [two-tailed test, voxel-level *P* < 0.01, corrected for multiple comparisons using Gaussian random field (GRF) correction, with cluster-level significance at *P* < 0.05].

**TABLE 2:** Brain regions with significantly different intra-network functional connectivity of RSNs between groups.

ICs	Brain regions	MNI coordinates			Brain networks	Peak *t*-values	Cluster size
		x	y	z			
**TAO > HC**							
17	Temporal_Inf_R	60	−30	−21	ECN	4.3481	48
12	Occipital_Mid_R	27	−99	3	VN	11.1452	3,312
**TAO < HC**							
6	Frontal_Sup_R	33	−9	60	DAN	−3.7815	43
27	Precuneus_L	0	−57	18	DMN	−3.9895	81
27	Frontal_Sup_Medial_L	−3	36	60	DMN	−3.9781	66
28	Cingulum_Post_L	−6	−51	30	DMN	−3.9779	107
17	Cingulum_Mid_L	−6	−39	36	ECN	−3.6501	55

RSNs, resting-state networks; ICs, independent components; MNI, Montreal Neurologic Institute; TAO, thyroid-associated ophthalmopathy; HC, health control; ECN, executive control network; VN, visual network; DAN, dorsal attention network; DMN, default mode network; R, right; L, left; Inf, inferior; Mid, middle; Sup, superior; Post, posterior.

### 4.4 Functional network connectivity analysis

**FIGURE 4:**
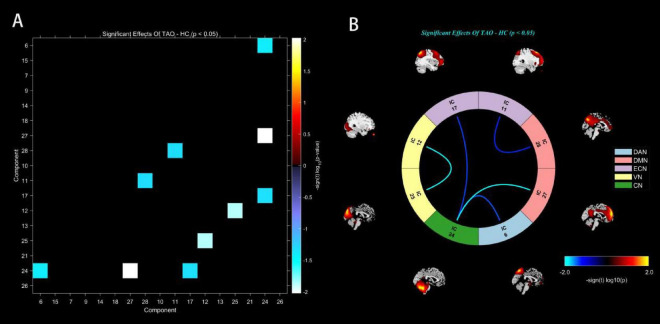
Matrix shows differences of internetwork functional network connectivity between two groups **(A)**. The CN-DAN, CN-DMN, ECN-DMN, CN-ECN and VN-VN connections were found to be significantly altered between two groups (*p* < 0.05) **(B)**. CN, cerebellum network; DAN, dorsal attention network; DMN, default mode network; ECN, executive control network; VN, visual network.

Significance and direction following two-sample *t*-tests (TAO-HC) on each pairwise correlation are depicted as the -sign(t)log10(*p*-value) (Figure 4A). FNC analysis showed decreased functional connectivity intra-VN and inter- CN and high-level cognitive networks (DAN, DMN, and ECN) between two groups (*P* < 0.05) (Figure 4B and Table 3).

**TABLE 3:** Significantly altered static functional network connectivity in TAO patients compared with HCs.

Brain networks	ICs	*t*-values	*p*-values
**TAO < HC**			
DAN-CN	IC6-IC24	−2.113	0.0388
CN-DMN	IC24-IC27	−2.681	0.0095
ECN-DMN	IC11-IC28	−2.020	0.0479
ECN-CN	IC17-IC24	−2.028	0.0470
VN-VN	IC12-IC25	−2.542	0.0136

TAO, thyroid-associated ophthalmopathy; HCs, health controls; ICs, independent components; DAN, dorsal attention network; CN, cerebellum network; DMN, default mode network; ECN, executive control network; VN, visual network.

### 4.5 Dynamic functional network connectivity analysis

#### 4.5.1 Cluster analysis

**FIGURE 5:**
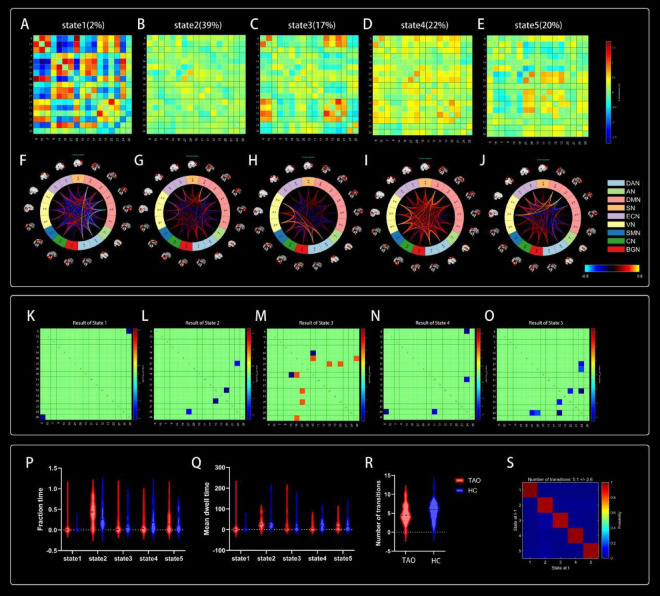
Cluster centroids for each state. Their percentage of total occurrences are listed above them **(A–E)**. Visualization of functional network connectivity at every state **(F–J)**. Significant differences in dFNC between TAO patients and HCs. The colored rectangles represent the dFNC between the two corresponding ICs, with warm colors indicating increased connectivity and cool colors indicating decreased connectivity **(K–O)**. Dynamic connectivity feature analysis for the TAO and HC groups **(P–S)**.

Five reoccurring states of dFNC matrixes were obtained throughout scans based on k-means clustering algorithm. The total percentages and visualization of the functional network connectivity of these five states in all subjects: State 1 (2%) (Figures 5A, F), State 2 (39%) (Figures 5B, G), State 3 (17%) (Figures 5C, H), State 4 (22%) (Figures 5D, I), and State 5 (20%) (Figures 5E, J). Among them, state 1 is characterized by modular connectivity and exhibits modular negative connectivity between DMN, ECN and DAN, AN. State 2 has sparse connectivity, state 4 has strong connectivity, while states 3 and 5 exhibit increased or weakened FC within and between certain networks.

### 4.6 Comparison of dFNC between groups

Two independent sample *t*-tests were used to further compare the dFNC matrix of each state between groups, and it was found that the dFNC of TAO patients was significantly different in states 1–5 compared to that of HCs (*p* < 0.01). Compared to HC, connectivity between DAN and BGN was reduced in the TAO group within state 1 (Figure 5K); brain networks with reduced dFNC in state 2 was intra-VN and CN-DMN (Figure 5L); brain networks with reduced dFNC in state 3 was SN-DMN (IC10-IC14), which also exhibited increased dFNC between SN-DMN (IC10-IC18), VN-DMN, and BGN-DMN (Figure 5M); state 4 exhibited decreased dFNC between DAN, ECN and CN (Figure 5N), while state 5 exhibited decreased connectivity between DMN, VN and CN as well as decreased intra-VN connectivity (Figure 5O and Table 4).

**TABLE 4:** Significantly different dFNC in states 1–5 between groups.

Brain networks	ICs	*t*-values	*p*-values
**State 1**			
DAN-BGN	IC6-IC26	−243.8	0.0026
**State 2**			
VN-VN	IC12-IC25	−3.324	0.0016
CN-DMN	IC24-IC27	−2.792	0.0072
**State 3**			
SN-DMN	IC10-IC14	−4.220	0.0002
SN-DMN	IC10-IC18	2.959	0.0057
VN-DMN	IC12-IC27	2.919	0.0063
VN-DMN	IC25-IC27	2.899	0.0066
BGN-DMN	IC18-IC26	2.845	0.0076
**State 4**			
DAN-CN	IC6-IC24	−3.440	0.0015
ECN-CN	IC11-IC24	−2.992	0.0050
**State 5**			
VN-VN	IC12-IC25	−3.061	0.0040
VN-CN	IC12-IC24	−3.780	0.0005
CN-DMN	IC24-IC27	−3.102	0.0036
CN-DMN	IC24-IC28	−2.722	0.0097

dFNC, dynamic functional network connectivity; ICs, independent components; DAN, dorsal attention network; BGN, basal ganglia network; VN, visual network; CN, cerebellum network; DMN, default mode network; SN, salience network; ECN, executive control network.

### 4.7 Comparison of dFNC temporal metrics between groups

Compared to HCs, the three dFNC temporal metrics [fraction of time (Figure 5P), mean dwell time (Figure 5Q), and number of transitions (Figure 5R)] and probability of transitions (Figure 5S) of TAO patients had no significantly difference in state 1–5 (Table 5).

**TABLE 5:** Differences in temporal features of dFNC states between TAO and HC groups.

Temporal features		*t*-values	*p*-values
FT	State 1	0.5497	0.5846
	State 2	0.9265	0.3579
	State 3	0.2470	0.8057
	State 4	−1.374	0.1744
	State 5	−0.2021	0.8405
MDT	State 1	0.5497	0.5846
	State 2	−0.3142	0.7544
	State 3	−0.1267	0.8996
	State 4	−1.1799	0.2427
	State 5	−0.5835	0.5618
NT		−0.8018	0.4259

TAO, thyroid-associated ophthalmopathy; HC, health control; FT, fraction time; MDT, mean dwell time; NT, number of transitions.

### 4.8 Support vector machine results

**FIGURE 6:**
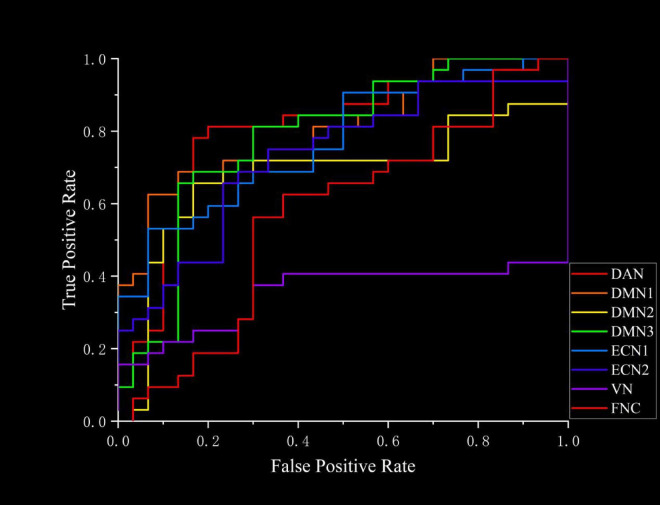
The ROC curves of SVM classifiers based on FC and FNC. ROC, receiver operating characteristic; SVM, support vector machine; FC, functional connectivity; FNC, functional network connectivity; DAN, dorsal attention network; DMN, default mode network; ECN, executive control network; VN, visual network.

The ROC curves generated by SVM for classifying TAO patients and HC based on FC and FNC are shown in Figure 6. Using FC as the classification feature, the area under the curve was 0.81667 (DAN), 0.81042 (DMN1), 0.68229 (DMN2), 0.78438 (DMN3), 0.77187 (ECN1), 0.7312 (ECN2), and 0.35208 (VN), with an accuracy of 75.81% (DAN), 77.42% (DMN1), 74.19% (DMN2), 75.81% (DMN3), 69.35% (ECN1), 70.97% (ECN2) and 51.61% (VN). Using FNC as a classification feature, the area under the curve was 0.57292 with an accuracy of 61.29% (Table 6).

**TABLE 6:** Classification performance of SVM based on FC and FNC.

Classification features	ICs	Brain regions	Accuracy	AUC
**FC**				
DAN	IC6	Frontal_Sup_R	0.7581	0.81667
DMN1	IC27	Precuneus_L	0.7742	0.81042
DMN2	IC27	Frontal_Sup_Medial_L	0.7419	0.68229
DMN3	IC28	Cingulum_Post_L	0.7581	0.78438
ECN1	IC17	Temporal_Inf_R	0.6935	0.77187
ECN2	IC17	Cingulum_Mid_L	0.7097	0.73125
VN	IC12	Occipital_Mid_R	0.5161	0.35208
FNC	–	–	0.6129	0.57292

SVM, support vector machine; FC, functional connectivity; FNC, functional network connectivity; ICs, independent components; AUC, area under the curve; DAN, dorsal attention network; DMN, default mode network; ECN, executive control network; VN, visual network; R, right; L, left; Inf, inferior; Mid, middle; Sup, superior; Post, posterior.

## 5 Discussion

The current study utilized ICA by integrating sFNC and dFNC to explore the aberrant functional network connectivity in individuals with TAO. The results indicated the following key findings: (1) Enhanced intra-network FC within the ECN manifested in the right inferior temporal gyrus and the VN in TAO patients compared to HC. Conversely, decreased intra-network FC was observed in the DAN, DMN, and the left middle cingulum of the ECN in TAO patients. (2) Analysis of sFNC uncovered notable reductions in intra-VN connectivity and connections from the CN to advanced cognitive networks (DAN, DMN, and ECN) in the TAO group. (3) Cluster analysis identified five recurrent states, each exhibiting significant alterations in dFNC, notably decreased intra-VN connections and links from CN to higher cognitive networks (DAN, DMN, and ECN), with compensatory increased connectivity between higher-level cognitive networks (DMN) and lower-level perceptual networks (VN and BGN). (4) Dynamic temporal metrics did not reveal significant discrepancies between the TAO and HC groups. (5) The FC-based SVM model demonstrated superior performance in discriminating TAO patients from healthy controls. These outcomes offer insights into the neural mechanisms underpinning central nervous system (CNS) symptoms, particularly cognitive impairments, in individuals with TAO.

Analyzing alterations in brain FC within RSNs assists in exploring the atypical intrinsic interactions within distinct spatial patterns in the brains of individuals with TAO (Beckmann et al., 2005; De Luca et al., 2006). Our study revealed a decrease in intra-network FC within the DAN, DMN, and ECN in individuals with TAO. The DAN, encompassing areas like the intraparietal sulcus, middle frontal gyrus, and frontal eye fields, plays a crucial role in top-down attentional orienting and the allocation of cognitive resources (Corbetta et al., 2008; Maidan et al., 2019). Previously, alterations in the DAN have been found in a variety of ophthalmic diseases such as early blindness (Ankeeta et al., 2021), neuromyelitis optica spectrum disorder (NMOSD) (Han et al., 2020), and glaucoma (Frezzotti et al., 2014; Li et al., 2017). One study found significantly lower ReHo values in parietal lobe and middle frontal gyrus in TAO patients (Jiang et al., 2021). An additional study identified disrupted brain function in the attention network among individuals with TAO, indicating a potential manifestation of underlying cognitive impairment (Chen et al., 2021). In line with these observations, our study demonstrated diminished intra-network functional connectivity in the DAN among individuals with TAO, which could be associated with cognitive dysfunction in this patient group. The DMN comprises the medial prefrontal cortex, posterior cingulate cortex, inferior parietal cortex, and precuneus (Raichle, 2015), which play important roles in working memory, cognition, and emotional processing (Spreng and Grady, 2010; Mantini and Vanduffel, 2013; Buckner and DiNicola, 2019). Zhu et al. (2022) identified anomalous spontaneous neuronal activity in the left posterior cingulate gyrus (LPCC) within the DMN of patients with TAO, suggesting a potential link to cognitive impairments. Quantitative susceptibility mapping (QSM) revealed a notable elevation in iron deposition within regions of the DMN in individuals with TAO (Hu et al., 2023). Multiple studies have reported alterations in gray matter thickness and volume among individuals with TAO, which are correlated with cognitive changes (Silkiss and Wade, 2016; Luo et al., 2022). In addition to these altered intra-network connections, there were abnormalities in the inter-network connections of the DMN. We found that functional network connectivity between the DMN and the ECN, CN was reduced in TAO patients. The ECN belongs to the advanced cognitive network. The CN is not only involved in motor control, but also performs important cognitive functions. Weakened functional connectivity between them may be related to cognitive decline in TAO patients. Based on these collective findings, our study revealed abnormal functional connectivity intra- and inter-network in the DMN of TAO patients, potentially impacting cognitive function in these patients. The ECN encompasses various frontal regions such as the dorsolateral prefrontal cortex, anterior cingulate, paracingulate in the medial frontal lobe, and posterior parietal cortex (MacDonald et al., 2000; Sridharan et al., 2008), and is involved in goal-directed stimulus and response selection, as well as cognitive control (Corbetta and Shulman, 2002; Seeley et al., 2007; Vincent et al., 2008). Silkiss and Wade (2016) found that the gray matter of the anterior cingulate was thinned in TAO patients. Wu et al. (2021) reported a significant decrease in the nodal properties of the anterior cingulate gyrus in the TAO group. In a somatic proton magnetic resonance spectroscopy (1H-MRS) investigation, a marked reduction in the choline/creatine (Cho/Cr) ratio was observed in the prefrontal cortex of individuals with TAO, suggesting that dysfunction in this region aligns with cognitive impairment (Bhatara et al., 1998). There is also a weakening of inter-network connectivity between the ECN and the DMN, CN, all of which are closely related to cognitive function. Therefore, we identified decreased functional connectivity within the ECN potentially indicating disturbances in executive and cognitive control in individuals with TAO.

Furthermore, our study revealed heightened intra-network functional connectivity in the VN and ECN, specifically the right inferior temporal gyrus. The VN is situated in the occipital lobe and plays a key role in processing visual information (Wang et al., 2008). A diffusion tensor imaging study showed increased FA values in the left middle occipital gyrus in patients with monocular amblyopia, which is thought to be a compensation of the healthy side for impaired vision (Li et al., 2013). Chen et al. (2021) found that the regional homogeneity (ReHo) values of the middle frontal gyrus and superior frontal gyrus, which constitute the ECN, were significantly higher, which was considered to indicate that TAO patients invoke additional neural resources in the prefrontal lobes to compensate for cognitive losses due to degenerative processes, representing a compensatory process in the early stages of cognitive impairment. Therefore, we speculate that the increase in FC within the VN and ECN in TAO patients is also a compensatory mechanism, i.e., coping with the decline in visual and cognitive functions by enhancing the functional activity of the relevant brain regions.

By static FNC analysis, we found that TAO patients had significantly lower connectivity between VN-VN and between CN and the high-level cognitive networks (DAN, DMN and ECN). Increasing neuroimaging evidence suggests that TAO patients have abnormal visual function. One study showed that the cerebral blood flow (CBF) value of the fusiform gyrus as well as the CBF/ALFF ratio were significantly decreased in TAO patients, and the decreased CBF/ALFF ratio was positively correlated with visual acuity (Chen et al., 2023). Another study showed that FA values in the middle occipital gyrus were decreased and radial diffusivity was increased in the TAO group (Wu et al., 2020). A brain voxel resting-state fMRI study found that fALFF values and ReHo values in the middle occipital gyrus were reduced in TAO patients compared with the HC group, reflecting reduced local brain activity in the occipital lobe and suggesting abnormal VN function (Chen et al., 2021). Combining these findings, we speculate that the reduced VN-VN connectivity in TAO patients is related to their abnormal visual function, and this abnormality may be caused by the reduced efficiency of functional integration between visually relevant regions within the VN The cerebellum was once thought to be involved only in motor control and coordination, but nowadays there is increasing evidence that the cerebellum also plays an important role in cognition, and its impairment may lead to abnormalities in thinking and emotions (Stoodley and Schmahmann, 2010; Schmahmann, 2019; Schmahmann et al., 2019). The DAN, DMN and ECN all belong to the high-level cognitive networks (Power et al., 2011; Guldenmund et al., 2016), which are responsible for a wide range of cognitively related brain functions. We found a general weakening of connectivity between the CN and these three high-level cognitive networks, implying an abnormal coordination and integration of higher cognitive functions involving the cerebellum, and thus a less efficient functional integration between higher cognitive networks, which may lead to cognitive impairment in TAO patients.

We identified five states that recurred over time through cluster analysis and found significant changes in dFNC in each state. Consistent with the results of the static FNC analysis, we found again that TAO patients had significantly reduced VN-VN as well as CN connectivity to the high-level cognitive networks (DAN, DMN, and ECN) in state 2, state 4 and state 5, and this recurrent result further confirms our speculation that visual and cognitive dysfunction exists in TAO patients. Additionally, the high degree of overlap between static and dynamic results suggests stability of functional network connectivity in TAO patients. Unlike the static results, we also found reduced CN-VN connectivity in state 5. The CN plays an important role in multimodal integration, containing afferent fibers of the visual sensory system that convey rich visual information and direct visual attention (Xiao and Scheiffele, 2018). Reduced CN-VN connectivity may suggest abnormal visual-motor integration (Xing et al., 2021). In contrast to the general decrease in inter-network connectivity in each of the other states, we observed enhanced connectivity between the DMN and the VN, BGN in the TAO group in state 3. The DMN belongs to the high-level cognitive network, whereas the VN and BGN belong to the low-level perceptual network, which plays a role in visual information processing and motor control, respectively (Graybiel et al., 1994; Desmurget et al., 2003; Lehéricy et al., 2006). We speculate that functional network reorganization may have occurred in the brains of TAO patients, allocating more cognitive resources to compensate for the impairment of visual and motor control functions by enhancing the interaction between high-level cognitive networks and low-level perceptual networks. In addition, we identified altered SN-DMN connectivity in TAO patients. The SN, which consists of the anterior insula and anterior cingulate cortex, is responsible for identifying the most relevant of internal versus external stimuli in order to guide behavior (Menon and Uddin, 2010), and is also involved in switching between the DMN and ECN (Sridharan et al., 2008). It has been previously shown that abnormal connectivity of the SN with the DMN and ECN is associated with cognitive impairment (Song et al., 2021). We hypothesized that altered SN-DMN connectivity in TAO patients may lead to abnormal switching between the DMN and ECN, causing misallocation of cognitive resources and thus cognitive impairment. We also examined three dynamic temporal metrics (fraction of time: percentage of the total number of FC windows in which a subject was in a given state; mean dwell time: the average amount of time a subject was in a state without switching to another state; number of transitions: the number of times a subject changed states) in TAO patients and found no significant differences between the TAO and HC groups. These three dynamic time metrics indicate the subject’s preference for the state and the stability of the state. No significant dynamic time changes were found in TAO patients compared to controls, suggesting that there may not be a decrease in state stability in TAO patients.

SVM is a machine learning algorithm utilized for classification tasks using neuroimaging data. A distinctive feature of SVM, unlike conventional univariate analysis methods for neuroimaging data, is its capability to be applied at the individual level, presenting high clinical translational efficiency (Orrù et al., 2012). In addition, classification by SVM to find potential neuroimaging signatures of disease is valuable for disease diagnosis. In this study, SVM was employed to differentiate between TAO patients and HCs using FC and FNC as classification features, respectively. The results indicated that the FC-based machine learning model exhibited superior performance in discriminating TAO patients from HCs, with accuracies ranging from 69.35 to 77.42% and areas under the curve spanning from 0.68229 to 0.81667. Notably, the FC within the visual network (VN) demonstrated poor classification results (accuracy 51.61%, area under the curve 0.35208). Conversely, FNC showed lower efficacy in distinguishing TAO from HC, with an accuracy of 61.29% and an area under the curve of 0.57292. Hence, we postulated that differences in FC within resting-state networks (RSNs) could potentially serve as a valuable neuroimaging indicator for distinguishing between TAO patients and healthy controls. Furthermore, some methods may be able to increase the accuracy of classification, such as increasing the number of subjects participating in the experiment, the use of more advanced classification models like deep learning models and the application of multimodal techniques.

There are some limitations of this study. Firstly, the sample size of our study was small, which is not conducive to the generalization of the findings, and the sample size should be enlarged for more in-depth studies in the future. Second, RSN values based on blood oxygen level-dependent signals are still affected by physiological noise such as heartbeat and respiratory activity. Third, the selection of the sliding-window length remains a subject of debate. We selected 30 TR as the window length based on the criterion that the minimum length should be more than 1/fmin. Finally, The varying disease stages among study participants could impact result consistency.

## 6 Conclusion

In summary, our research highlighted substantial alterations in functional connectivity within RSNs, as well as in sFNC and dFNC in individuals with TAO, potentially linked to visual and cognitive deficits. Notably, the dynamic temporal metrics in the TAO group did not significantly differ from those in the HC group. Furthermore, distinctions in functional connectivity within RSNs could potentially serve as a neural biomarker for discriminating TAO patients from healthy controls. These findings provide valuable insights into the underlying neural mechanisms associated with visual and cognitive impairments in individuals with TAO.

## Data Availability

The raw data supporting the conclusions of this article will be made available by the authors, without undue reservation.

## Footnotes

1
http://www.rfmri.org/dpabi

2
http://www.fil.ion.ucl.ac.uk

3
http://icatb.sourceforge.net/
